# Interplay of Developmental Hippo–Notch Signaling Pathways with the DNA Damage Response in Prostate Cancer

**DOI:** 10.3390/cells11152449

**Published:** 2022-08-07

**Authors:** Ioanna Mourkioti, Andriani Angelopoulou, Konstantinos Belogiannis, Nefeli Lagopati, Spyridon Potamianos, Efthymios Kyrodimos, Vassilis Gorgoulis, Angelos Papaspyropoulos

**Affiliations:** 1 Molecular Carcinogenesis Group, Department of Histology and Embryology, Medical School, National Kapodistrian University of Athens (NKUA), 11527 Athens, Greece; 2 Biomedical Research Foundation, Academy of Athens, 11527 Athens, Greece; 3 First ENT Department, Hippocration Hospital, University of Athens, 11527 Athens, Greece; 4 Clinical Molecular Pathology, Medical School, University of Dundee, Dundee DD1 9SY, UK; 5 Molecular and Clinical Cancer Sciences, Manchester Cancer Research Centre, Manchester Academic Health Sciences Centre, University of Manchester, Manchester M20 4GJ, UK; 6 Center for New Biotechnologies and Precision Medicine, Medical School, National and Kapodistrian University of Athens, 11527 Athens, Greece; 7 Faculty of Health and Medical Sciences, University of Surrey, Surrey GU2 7YH, UK

**Keywords:** prostate cancer, DNA damage response (DDR), Hippo pathway, Notch pathway, interplay

## Abstract

Prostate cancer belongs in the class of hormone-dependent cancers, representing a major cause of cancer incidence in men worldwide. Since upon disease onset almost all prostate cancers are androgen-dependent and require active androgen receptor (AR) signaling for their survival, the primary treatment approach has for decades relied on inhibition of the AR pathway via androgen deprivation therapy (ADT). However, following this line of treatment, cancer cell pools often become resistant to therapy, contributing to disease progression towards the significantly more aggressive castration-resistant prostate cancer (CRPC) form, characterized by poor prognosis. It is, therefore, of critical importance to elucidate the molecular mechanisms and signaling pathways underlying the progression of early-stage prostate cancer towards CRPC. In this review, we aim to shed light on the role of major signaling pathways including the DNA damage response (DDR) and the developmental Hippo and Notch pathways in prostate tumorigenesis. We recapitulate key evidence demonstrating the crosstalk of those pathways as well as with pivotal prostate cancer-related ‘hubs’ such as AR signaling, and evaluate the clinical impact of those interactions. Moreover, we attempt to identify molecules of the complex DDR–Hippo–Notch interplay comprising potentially novel therapeutic targets in the battle against prostate tumorigenesis.

## 1. Introduction

Prostate cancer is the second leading cause of cancer death in men in the USA and a major cause of cancer morbidity and mortality in men worldwide, resulting in around 34,500 deaths in 2022 in the USA [1]. The prostate is a gland of the male reproductive system that produces semen and requires androgens for its normal growth and function. Androgens are sex hormones responsible for the development and maintenance of the male reproductive system and secondary sexual characteristics [2]. In men, the most abundant androgens are testosterone and dihydrotestosterone (DHT). Apart from regulating prostate growth and normal function, androgens also contribute to prostate tumorigenesis by binding and activating the androgen receptor (AR), a nuclear receptor expressed in prostate cells [3]. Activation of the androgen receptor promotes the expression of specific genes that drive prostate cell growth [4].

At an early stage, almost all types of prostate cancer are androgen-dependent and require the AR signaling pathway for their survival. As a result, androgen deprivation therapy (ADT) holds a central place in early-stage disease management and involves a reduction in testosterone levels produced by the testicles, achieved either by surgical or medical castration [5]. Medical castration represents the most preferable strategy for ADT due to its possible reversibility [6]. Medical castration options include traditional luteinizing hormone-releasing hormone (LHRH) agonists and the gonadotropin-releasing hormone (GnRH) antagonist degarelix [6]. Notably, both options are associated with an increase in cardiovascular risk and potential differences may be attributed to the different mechanisms through which LHRH agonists and degarelix suppress testosterone levels [7]. Data obtained by randomized trials provide a possible cardiovascular benefit for degarelix versus LHRH agonists, but further research is required to conclusively determine safety [7]. ADT is not curative and disease progression may occur following 1–3 years after ADT. In most cases, the reason for this is that cancer cell pools manage to survive and continue to proliferate while becoming androgen-independent, and this stage is therefore called castration-resistant prostate cancer (CRPC) [8]. CRPC is accompanied by significantly low patient survival rates [9,10]. It is, thus, of critical importance to shed light on the molecular mechanisms underlying resistance to ADT and progression to CRPC, as this will guide new therapeutic approaches.

The DNA damage response (DDR) pathway is a major pathway of the genetic landscape of prostate cancer. The DDR pathway is charged with preserving the integrity of the genome, and is hierarchically structured in a signaling cascade involving DNA damage sensors, signal mediators and transducers as well as effectors to impose a global cellular response by recruiting the most suitable DNA repair molecules [11,12,13]. Based on various preclinical and clinical studies, many alterations of the DDR pathway are accumulated during prostate cancer progression to CRPC, which can induce resistance to therapy [14].

Another pathway of major significance in the prostate cancer field is the Hippo pathway, a major developmental pathway which was originally discovered in *Drosophila melanogaster* [15], but later emerged as an evolutionarily conserved regulator of organ size, proliferation and stem cell biology across species. This signaling pathway is a cascade of kinase enzymes activated by a variety of signals involved in cell–cell contact, nutrient abundance, cellular stress and interaction with the microenvironment, resulting in the inhibition of the YAP/TAZ downstream effector proteins. In the prostate cancer field, this pathway can be deregulated by various different mechanisms, justifying why this pathway may be an important candidate for targeted therapy [16].

The Notch signaling pathway is another critical pathway actively involved in the development, progression and metastasis of prostate cancer [17]. The Notch pathway has been shown to regulate the tumorigenicity of prostate cancer stem cells, thereby modulating oncogenic proliferation as well as resistance to chemotherapy. As a result, the Notch signaling pathway has already been highlighted as a therapeutic target for the elimination of prostate cancer stem cells [18].

In this review, we aim to recapitulate recent findings on how the DDR, Hippo and Notch signaling pathways contribute to prostate tumorigenesis, particularly elaborating on crosstalk mechanisms among them. We additionally attempt to summarize how elements of the complex DDR–Hippo–Notch interplay may constitute targets of contemporary and novel therapeutic approaches against the various levels of prostate cancer development.

## 2. The DDR Pathway in Prostate Cancer

**Figure 1:**
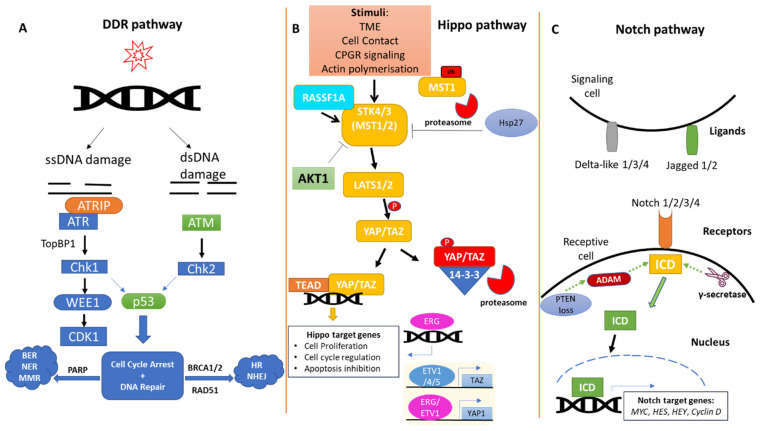
**Schematic representation of the DDR, Hippo and Notch signaling pathways in prostate cancer.** (**A**)**.** Mutations in DDR genes including *ATM*, *TP53*, *BRCA1/2* and *RAD51* are likely implicated in prostate carcinogenesis. The DDR pathway is largely conserved among different types of human cancer. (**B**)**.** The Hippo kinase cascade is actively involved in prostate cancer. One of the routes through which YAP activity may be affected in prostate cancer is via MST1 kinase ubiquitination and subsequent proteosomal degradation, elicited by the molecular chaperone HSP27. Upon MST1 degradation, YAP translocates to the nucleus where it resumes its transcription coregulator activity. ETS factors and ETS-regulated genes, such as ERG, also have an important role in YAP and TAZ regulation. Especially ERG, apart from regulating the expression of YAP1, it can also bind to YAP1/TEAD binding sites and transactivate Hippo target genes. (**C**)**.** The Notch pathway is deeply involved in prostate tumorigenesis. When a Notch ligand (Delta-like 1/3/4 or Jagged-1/2) binds to Notch transmembrane receptors (Notch 1/2/3/4), the receptors are cleaved by members of Metalloprotease (ADAM) family and γ-secretase. This cleavage releases the Notch intracellular domain (NICD), which translocates to the nucleus, where it regulates the expression of genes including *MYC, HES, HEY, Cyclin D,* etc.

Human cells are continuously exposed to extrinsic (e.g., genotoxic agents) and/or intrinsic (e.g., free radicals as a metabolic byproduct) insults that often affect the integrity of important biomolecules, such as proteins and DNA. In response to accumulation of DNA mutations and genomic instability, cells activate DNA damage response (DDR) pathways in an attempt to maintain their integrity. DDR pathways are composed of sensors and effector molecules that detect and repair DNA lesions through damage-specific repair modules. These can be classified depending on the type of DNA damage (single- vs. double-strand break repair) and the efficacy of the repair (error-free, error-prone) [19,20,21] (Figure 1A).

DDR mechanisms are generally conserved among different cancer types, and their activation mostly depends on the type of DNA damage. For single-strand break (SSB) DNA lesions, the cell employs two error-free operating systems, the base excision repair (BER) system for small base alterations and the nucleotide excision repair (NER) system for the removal of an extremely broad range of helix-distorting base lesions [22]. A third module involved in SSB lesions is the mismatch repair (MMR) system which improves replication fidelity by removing mismatched bases during replication [23]. Double-strand break (DSB) lesions are reversed by the error-free homologous recombination repair (HRR) pathway or the error-prone repair pathway called non-homologous end joining (NHEJ) [21,24].

Many proteins are involved in the various DDR pathways and each one carries out a different role in the process. Ataxia telangiectasia and Rad3-related (ATR) and Ataxia–telangiectasia-mutated (ATM) kinases, as well as DNA-dependent protein kinases (DNA-PKs), are effector proteins responsible for the timely resolution of single-strand and double-strand DNA lesions, respectively. These proteins cooperate with checkpoint kinases to arrest cell-cycle progression by reducing the activity of cyclin-dependent kinases to allow time for the repair process to take place [19]. If DNA damage is irreparable, cell death/apoptosis or senescence are triggered in order to avoid the propagation of potentially harmful cells. However, under specific circumstances, dysplastic cells continue to proliferate, thus establishing a precancerous state, which may ultimately progress to cancer [25].

In the case of prostate cancer, many components of the DDR pathway have been reported to be defective, in both primary and advanced stages [26]. Those defects pertain to germline and, more commonly, somatic cells (8% versus 23%, respectively) [27,28]. Regarding germline aberrations, *BRCA2* mutations are more prevalent in metastatic than primary disease, accounting for 11.8% of metastatic and 4.6% of primary cases [29]. In terms of somatic DDR alterations, *TP53* mutations are detected in 3–20% of prostate tumors at the time of diagnosis (primary disease) [30,31] and in up to 42% of advanced prostate tumors [32,33], correlating with high-grade disease, cancer relapse, castration resistance and metastasis [34,35]. According to a separate report, mutations in key DDR genes were reported in approximately 10% of the cases in primary tumors, whereas DDR genes were altered in 27% of metastatic cases, confirming that advanced stages of prostate cancer correlate with increasingly higher incidence of mutations in those genes [36]. In keeping with the above observations, specific DDR gene mutations are more frequent in metastatic CRPC than primary disease, as also found by Grasso et al. demonstrating that *BRCA2*, *ATM* and *RAD50* are mutated in 46% of metastatic compared to 27% of localized disease [37]. In general, CRPC patients are characterized by an increased prevalence of genomic mutations in *TP53* (53%), *RB1* (21%), the PTEN-PI3K pathway (49%) and *AR* (63%) compared to localized disease [28,38]. Those mutation rates confirm the notion that although primary, locally advanced and metastatic prostate tumors often share defects in the same key DDR players, concomitant mutations in DDR genes significantly increase upon disease course, culminating in a progressively impaired DDR.

## 3. The Hippo Pathway in Prostate Cancer

The tumor suppressor Hippo pathway is a highly conserved regulator of organ size and cell fate and modulates cell proliferation and apoptosis [39] (Figure 1B). Disruption of the Hippo pathway is observed in multiple cancer types and occurs both at the genomic and post-translational levels. Potentially owing to its important role in early embryogenesis, inactivating mutations of individual components within the kinase cascade are rare and mainly somatic [40,41,42]. Briefly, upon activation of the Hippo pathway, STK4/3 (MST1/2) kinases phosphorylate LATS1/2 kinases along with the adaptor protein MOB1, in response to various stimuli. In turn, LATS1/2 phosphorylate YAP/TAZ proteins, which remain in the cytoplasm in complex with chaperone 14-3-3 proteins, and are subsequently targeted for proteasomal degradation [43]. The YAP/TAZ transcriptional role is manifested in their unphosphorylated state, where they translocate into the nucleus and conjugate with transcription factors such as TEAD to stimulate the transcription of genes involved in cell proliferation, cell-cycle regulation, inhibition of apoptosis, migration and invasion [16].

Disruption of the Hippo pathway has been linked to prostate cancer during both initiation and metastatic stages [44] (Figure 1B). Additionally, components of the Hippo pathway have been established as modulators of stem cells and embryonic development, and cancer stemness, particularly in the prostate context, has been reported to be Hippo-regulated [45]. The transcriptional co-activator YAP1 and its paralog TAZ have been found to be overexpressed in prostate cancer, establishing a pro-tumorigenic phenotype which is attenuated upon YAP/TAZ knockdown [46,47].

YAP/TAZ expression may be regulated by transcription factors belonging to the E26 transformation-specific (ETS) family and YAP/TAZ are considered to be drivers of prostate carcinogenesis [48,49]. Specifically, ETS-regulated gene (ERG) proteins can directly bind to the YAP1 promoter, regulating its expression, or transactivate Hippo target genes by occupying the YAP1/TEAD binding sites [48]. YAP expression can also be induced by ETV1, a member of the ETS protein family, fostering the initiation of prostate malignancy when cooperated with lysine-specific demethylase 4A (KDM4A, also known as JMJD2A) [49]. Moreover, the ETS factors ETV1/4/5 are necessary for TAZ transcription in prostate cancer cells, leading to the expression of SH3 domain-binding protein 1 (SH3BP1), which drives cell motility and constitutes a direct target of TAZ [50].

Beyond ETS-mediated regulation, YAP activity might be indirectly regulated by heat-shock protein 27 (HSP27), a molecular chaperone contributing to the degradation of MST1 by the proteasome. This way, the phosphorylation of the downstream factors LATS1 and MOB1 is decreased, favoring the nuclear transport and transcription activity of non-phosphorylated YAP [51]. Of note, MST1 degradation promoted through its physical interaction with HSP70 mediates cisplatin resistance in prostate cancer cells [52]. MST1 has also been reported to be suppressed by miR-18a [53] or downregulated via epigenetic silencing mediated by c-Myc and EZH2 in prostate cancer [16]. It has also been shown that MST1 may directly interact with and inhibit the AKT1 serine/threonine kinase in human prostate cancer cells [54]. As the disease progresses from androgen-dependent to castrate-resistant, a decrease in both MST1 and PTEN tumor suppressor levels enhances AKT1 activity, thus linking MST1 loss with the phosphoinositide 3-kinase/protein kinase B (PI3K/AKT) pathway activity in driving disease progression [26,54].

YAP may also be regulated by the tumor suppressor LKB1 via the MAPK and Hippo kinases leading to YAP phosphorylation [55]. It has been found that LKB1 mutations, particularly in conjunction with *PTEN* heterozygosity, may promote lethal metastatic prostate cancer [56]. Hence, the linkage between LKB1 and the Hippo pathway could provide significant mechanistic insight regarding the treatment of LKB1-mutated cancers.

The role of the extracellular matrix (ECM) as an essential component of the tumor microenvironment and its interplay with tumor cells, including prostate cancer cells, has proven important in facilitating metastasis [57]. YAP/TAZ proteins are involved in cell-matrix adhesion and mechanotransduction signals, serving as sensors of ECM composition and activated by ECM stiffness, which is usually increased in tumors, including prostate cancer [58,59,60]. Metastasis may be promoted in tumor cells by manifesting resistance to a type of programmed cell death, known as anoikis, which is normally triggered when the cells detach from the matrix. Anoikis is mediated by the activation of LATS1/2 kinases and subsequent YAP inhibition [61]. Additionally, a3b1 integrin is able to restrain prostate cancer metastasis by regulating YAP/TAZ activity, and interestingly, stromal reprogramming promoted by the tumor may enhance prostate cancer metastasis by producing tenascin C via inhibition of YAP/TAZ [62].

All the above, in combination with the genetic variability identified in Hippo factors and associated with different clinical outcomes of prostate cancer, indicate the active role of the pathway throughout prostatic carcinogenesis and underline the significance of the potential exploitation of Hippo factors as biomarkers of disease prognosis and therapeutic targets [63].

## 4. The Notch Pathway in Prostate Cancer

The Notch pathway has an essential role in regulating cell proliferation, fate, differentiation and stem cell maintenance (Figure 1C). The Notch signaling pathway has been shown to be fundamental to normal prostate development, and Notch deregulation is involved in prostatic carcinogenesis by affecting the response to hormone-based treatments [64,65]. When a Notch ligand (Delta-like 1/3/4 or Jagged-1/2) binds to Notch transmembrane receptors (NOTCH 1/2/3/4), the receptors are cleaved by members of the A disintegrin and metalloprotease (ADAM) family and γ-secretase. This proteolytic cleavage results in the release of the Notch intracellular domain (NICD) fragment, which translocates to the nucleus, where it can directly regulate gene transcription [42,66].

Upregulation of Notch signaling has been observed in malignant human and mouse prostate cells [17,67]. When Notch is overexpressed, the migratory and invasive potential of prostate cancer cells may increase as a result of epithelial to mesenchymal transition (EMT) [68]. One of the four known Notch receptors, NOTCH1, has been found to be highly upregulated in malignant prostate cells compared to normal counterparts [69]. Loss of NOTCH1 is able to reduce the oncogenic potential of prostate cancer cells, by compromising their ability to form tumor spheres and metastasize [69]. In line with this, gamma secretase inhibitors (GSI), which prevent Notch signaling at the receptor level, can reduce the proliferative capacity of prostate cancer cells, and tumors formed in immunocompromised mice by prostate cancer xenografts were significantly impaired upon NOTCH1 loss or treatment with GSI [69].

Notch signaling has been reported to become activated in prostate cancer cell lines exhibiting resistance to the clinically available AR inhibitor enzalutamide, as verified by the increased levels of cleaved NOTCH1, HES1 and c-MYC [70]. It has been suggested that the Notch pathway likely contributes to resistance to enzalutamide in prostate cancer, which can be reversed by Notch signaling inhibition [70]. There is also evidence that inhibition of NOTCH1 combined with antiandrogens, including enzalutamide or abiraterone, can efficiently attenuate tumor growth and prevent metastasis in CRPC [69]. In addition, Notch targeting via GSI may also act synergistically with the microtubule stabilizer docetaxel, by increasing its anti-cancer effect [71], as observed mainly in prostate cancer stem cells. Those results holds great promise in the development of therapeutic approaches targeting cancer stem cell pools.

Although most of the links between Notch receptors and prostate cancer involve NOTCH1, NOTCH2 was also found increased in several prostate cancer cell lines, with the highest levels expressed in the most aggressive ones, whereas NOTCH3/4 were not detectable [72]. Notch ligands are also differentially expressed among metastatic prostate cell lines. There seems to be an association between the Notch ligand DLL1 and androgen responsiveness, as DLL1 levels are increased in androgen-dependent cell lines compared to androgen-independent ones [73]. The Notch ligand Jagged-1 was upregulated mostly in metastatic prostate cells, and this was correlated with tumor recurrence, whereas no significant differences in Jagged-2 levels were detected [74]. These findings suggest the potent contribution of DLL1 and Jagged-1 in prostate cancer progression, but further investigation is required. Of note, DLL3 has been correlated with aggressive phenotypes and is mainly expressed in castration-resistant neuroendocrine prostate tumors [75].

Although the mechanistic aspects of Notch activation in prostate cancer have not been fully elucidated yet, it has been shown that Notch activation is able to promote metastasis in *Pten*-null mouse prostate cancer [76]. The Notch pathway has also been suggested to alter the prostate tumor microenvironment [77]. Particularly, Jagged-1 upregulation has been found to promote the formation of reactive stroma in *Pten*-null mice [77]. Along those lines, PTEN deficiency may activate the Notch signaling pathway in prostate tumors [78]. Specifically, there is evidence that PTEN loss is able to promote ADAM17 upregulation in prostate cancer, which functions as a Notch pathway activator [78]. Notably, inhibition of the Notch pathway is able to trigger senescence in PTEN-deficient advanced prostate cancer, a finding which deserves to be therapeutically explored [78].

## 5. Interplay of DDR/Hippo/Notch Pathways in Prostate Cancer

**Figure 2:**
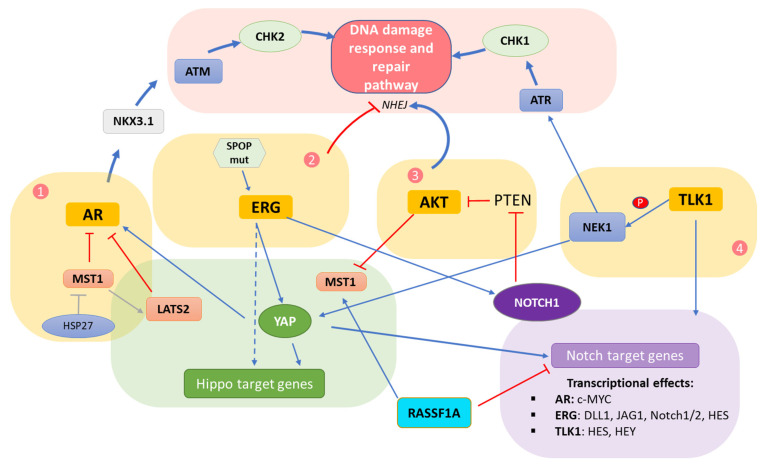
**DDR/Hippo/Notch pathway interplay in prostate cancer.** Schematic representation of the DDR/Hippo/Notch pathway crosstalk in prostate cancer through the AR, ERG, AKT and TLK1 molecular hubs (1–4) boxed in yellow. Stimulatory effects between different pathway components are represented with blue lines while inhibitory signals are marked in red.

CRPC is accompanied by poor prognosis, in part due to the lack of an effective therapeutic approach. It is, therefore, of critical importance to shed light on the molecular mechanisms underlying progression to castration resistance. In this section, the complex interplay of the three signaling pathways–Hippo/Notch/DDR—will be discussed in the context of prostate cancer. Four molecular ‘hubs’ have been identified as pivotal intersection points, and those involve the androgen receptor (AR), the oncoprotein ERG, AKT kinase in the PI3K/AKT pathway and the TLK1/NEK1 axis. Key molecular events of the complex interplay among the DDR/Hippo/Notch pathways within each ‘hub’ are depicted in Figure 2.

### 5.1. Crosstalk Involving the AR

The AR plays a crucial role in the development of male reproductive tissues and its main anabolic effects are mediated by testosterone and dihydrotestosterone (DHT) [79]. In the absence of a ligand, the AR remains in the cytoplasm in association with heat-shock and other chaperone proteins such as HSP90. Upon ligand binding, the AR undergoes conformational changes, dissociating from HSP90 following PKA-mediated phosphorylation, which enables AR binding to HSP27, a critical step for its nuclear transport [80]. Inside the nucleus, the AR dimerizes and binds to androgen responsive elements (AREs) to modulate gene transcription. AR transcriptional activity is regulated by specific proteins that associate with the AR either positively (co-activators) or negatively (co-repressors) [81]. This differential AR-mediated transcriptional response is achieved via chromatin reorganization and histone modifications [80,82].

Recent studies have shown that AR activity may be regulated by the physical interaction of the AR with key Hippo pathway factors, including YAP1 or LATS2, leading to positive and negative regulation of the AR, respectively [83,84]. It was shown that knockdown of MST1 increased YAP1 nuclear localization and YAP1-AR interactions in prostate cancer cells, which correlated with augmented cell growth independent of androgen exposure, whereas expression of ectopic MST1 had the opposite effect [83]. Of note, YAP1 activation resulted in higher levels of c-MYC (a Notch pathway target gene), which in turn, upregulated AR transcription and enhanced the stability of the full-length AR and its splice variants [85].

In keeping with the above observations, the HSP27 chaperone has been found to promote the proteasomal degradation of MST1, which results in impaired activation of the MST/Hippo pathway kinase cascade [51]. HSP27 is upregulated in prostate cancer, in particular in response to ADT, and patients with high levels of HSP27 display poor prognosis [16]. Interestingly, another study has demonstrated that MST1 may directly associate with the AR leading to reduced AR transcriptional activity [86]. Given that HSP27 also forms a complex with the AR, it could be possible that a trimeric AR–HSP27–MST1 complex is formed to inhibit AR transcriptional activity; however, this has not been directly tested yet [86].

A body of evidence has additionally highlighted the implication of the AR in DDR regulation during prostate cancer progression. Bowen et al. demonstrated that the AR activates the DDR pathway through stimulation of ATM by the AR target gene NKX.3.1 [87]. Interestingly, the Hippo pathway scaffold RASSF1A has been shown to be directly activated by ATM upon irradiation (IR) or other sources of DNA damage, leading to apoptosis [88,89,90]. However, a functional link between the AR and RASSF1A has not yet been identified.

Activation of the AR by DHT leads to transcriptional upregulation of DNA repair genes involved in various repair pathways. More specifically, the AR pathway directly regulates the NHEJ factor DNA-dependent protein kinase catalytic subunit (DNA-PKcs) resulting in a slight increase in NHEJ activity upon androgen addition [91]. Additionally, IR treatment caused a marked induction of the androgen target genes *TMPRSS2* and *FKBP5* in prostate cancer cells, suggesting that DNA damage may directly induce AR activity [14]. In that regard, AR signaling was found to promote the fusion of two genes, *TMPRSS2* and *ERG*, by inducing chromosomal rearrangements in both gene loci, following exposure to genotoxic, DSB-inducing agents [92]. The TMPRSS2–ERG fusion is uniquely present in prostate cancer in approximately 50% of the cases; however, it does not occur upon lack of AR signaling [92]. Overall, the above findings support the notion that developmental pathways, such as the Hippo and Notch pathways, may actively regulate AR signaling, thereby modifying cellular response to DNA damage.

### 5.2. Crosstalk Involving the ERG Oncoprotein

Gene fusions have a critical role in cancer onset and progression [93]. In the case of prostate cancer, the TMPRSS2–ERG fusion leads to upregulation of the ETS transcription factor ERG [94], which drives invasion and migration of prostate cancer cells and restricts their differentiation capacity [95]. The TMRSS2–ERG gene fusion is considered an early event in the development of prostate cancer, and it often coexists with *PTEN* deletion to drive frank carcinoma in around 20% of primary and 50% of advanced prostate tumors [44,94]. Importantly, the TMRSS2–ERG fusion has been shown to inhibit NHEJ and increase cellular markers of DNA damage (such as γH2AX and 53BP1 foci) by inhibiting DNA-PKs [96].

In prostate cancer, loss of the tumor suppressor PTEN increases ERG levels by inhibiting PTEN-mediated degradation of ERG [97,98] and resulting in a cascade of events that induce cell proliferation through the Hippo pathway. Mechanistically, PTEN loss allows ERG to induce YAP1 expression and promote YAP-dependent gene transcription, whereas ERG knockdown results in a decrease in YAP1 protein levels [44,48]. When YAP1 activation is induced in vivo by ETV1, it causes prostatic intraepithelial neoplasia (PIN) lesion formation, which when combined with a single copy loss of *PTEN*, progresses to malignant carcinoma [49]. In addition, PTEN is a negative regulator of the PI3K/AKT pathway, which has been shown to promote oncogenic proliferation of prostate cells at the expense of apoptosis [99].

There is also clear evidence that the expression of Notch genes—specifically, NOTCH1 and NOTCH2 receptors—can be positively regulated by ERG in prostate tumors [100]. ERG-dependent NOTCH1/2 upregulation promotes EMT in prostate cancer cells, which can be reversed upon ERG and NOTCH1/2 silencing [100]. In accordance with this, the Notch pathway has been found activated in ERG-inducible LNCaP *TMPRSS2:ERG* (T2E) prostate cancer cells, as demonstrated by the upregulation of direct Notch pathway target genes such as HES1 [101]. Thus, the ERG oncoprotein serves as a critical entity converging Hippo and Notch pathway activities during prostate carcinogenesis.

Speckle- type BTB/POZ protein (*SPOP*) mutations represent the most frequently identified type of genomic mutations in prostate cancer [102]. These mutations lead to elevated ERG levels in prostate cancer, through decreased ERG degradation. Specifically, SPOP functions as a cullin 3-based E3 ubiquitin ligase involved in the ubiquitination and proteasomal degradation of ERG in prostate tumors [94]. Hence, the inability of mutated SPOP to interact with ERG leads to full-length ERG accumulation in prostate cancer [94]. Interestingly, the *TMPRSS2–ERG* fusion mostly encodes truncated ERG proteins, which also appear to be resilient to SPOP-mediated proteasomal degradation [103].

Notably, SPOP mutations have been linked to genomic instability promoted through the DDR pathway in prostate cancer [102]. Specifically, DSBs induced by IR or the topoisomerase inhibitor camptothecin (CPT) become repaired via NHEJ, whereas homology directed repair (HDR) is impaired upon SPOP loss or mutation in prostate cancer cells [104]. It has been suggested that SPOP mediates the transcription of repair factors, including BRCA2, ATR, CHK1 and RAD51, and is, therefore, necessary for the recovery of stalled replication forks [105]. Interestingly, it was recently shown that prostate cancer cells harboring SPOP mutations are sensitive to ADT [106], an observation which warrants further investigation.

DNA damage caused by genotoxic agents, such as docetaxel, CPT and IR, may lead to the degradation of normal ERG and TMPRSS2–ERG proteins in a SPOP-independent manner, suppressing prostate cancer [98]. Specifically, ERG double phosphorylation by glycogen synthase kinase 3 beta (GSK3β) and the DNA damage-induced ATR-CHK1-WEE1 axis has been found to elicit the SPOP-independent proteasome-mediated destruction of ERG [98]. However, this DNA damage-induced ERG destruction seems to be abrogated by PTEN or GSK3β inactivation [98]. Since Notch is regulated by ERGs, the inefficient degradation of ERGs in a PTEN-null environment could possibly be one of the mechanisms leading to tumor metastasis mediated by Notch, as identified in a prostate-specific Pten-null mouse model [76]. Moreover, it was recently revealed that truncated ERGs likely form positive feedback loops with wild-type SPOP, thus increasing its levels [106].

Importantly, it should be mentioned that ERG fusions and SPOP mutations are mutually exclusive in prostate cancer and manifest common gene signatures, highlighting the potentially redundant role of these two genomic events. Taken together, it is evident that the TMPRSS2–ERG fusion holds a central role in the onset and progression of prostate cancer, with ERG serving as a pivotal hub for DDR/Hippo/Notch pathways, all actively implicated in prostate cancer pathophysiology.

### 5.3. Crosstalk within the AKT Hub

Another junctional point where the DDR/Notch/Hippo pathways may directly or indirectly intersect is the serine/threonine AKT kinase. Accumulating evidence suggests the active implication of AKT in prostate cancer, especially upon disease progression [99]. The AKT family of serine/threonine protein kinases plays a key role in various aspects of cellular biology, including proliferation, survival, metabolism and tumorigenesis [107].

Several recent studies have involved AKT in regulating the cell-cycle G1/S and G2/M transition checkpoints, DNA damage responses and genomic stability [108]. High AKT activity is capable of inhibiting the ATR/CHK1 pathway as well as HRR, either directly through CHK1 phosphorylation or indirectly by hampering the recruitment of DSB sensor players (such as BRCA1 and RAD51) to lesion sites [108]. On the other side, AKT can trigger the DDR pathway by activating NHEJ while responding to DSBs in a DNA-PK- or ATM/ATR-dependent fashion [108]. As a result, tumors exhibiting high levels of AKT, as in prostate cancer [109,110], may be prone to genomic instability upon loss of checkpoint and/or HRR proficiency, which underlines the important link between the DDR and AKT.

Moreover, Notch and Hippo signaling are also interconnected with AKT in prostate cancer. It has been shown that MST2 may be directly phosphorylated by AKT, which inhibits MST2 association with its upstream activator RASSF1A, thereby repressing MST2 kinase activity [111]. Alternatively, AKT signaling may promote MST2 interaction with its inhibitor RAF-1, resulting in inhibition of MST2 caspase-mediated cleavage which produces its active form [111]. Since PTEN loss is observed in a large proportion of prostate tumors resulting in enhanced AKT activity [112], Hippo pathway inactivation via AKT is a likely event in the context of prostate cancer, which remains to be deeply explored. Along the same lines, upregulation of NOTCH1 was shown to lead to PTEN inactivation, thereby reciprocally augmenting AKT, which, in turn, activates the Notch pathway via ADAM17 [78].

### 5.4. Crosstalk Involving TLK/NEK

Tousled-like kinase 1 (TLK1) and 2 (TLK2) are members of the tousled-like kinase family of serine/threonine kinases, have similar substrates and both of them participate in the DNA repair and replication process [113]. More specifically, TLK1 plays an important role in DNA replication and transcription and cell-cycle checkpoint regulation, as well as DNA damage response and repair pathways [114,115,116]. As shown by Singh et al., TLK1/1B regulates DDR through NIMA-related kinase 1 (NEK1), as it binds and phosphorylates NEK1 upon DNA damage [117]. In response to genotoxic stress, NEK1 translocates to the nucleus where DNA damage foci are formed, and cell-cycle checkpoints are triggered through activation of CHK1 and/or CHK2 [118,119]. The crucial role of NEK1 in DDR regulation is strengthened by the finding that NEK1 is essential for ATR-CHK1 activation [120].

Upon development of castration-resistance, prostate cancer growth is, at least in part, achieved through mTOR activation and consequent upregulation of TLK1B [121]. The molecular mechanism was explored by Khalil and Benedetti who found that ADT inhibits the AR signaling pathway by blocking the nuclear relocation of AR, resulting in downregulation of the androgen-responsive gene *FKBP5*, which controls intracellular glucocorticoid signaling and holds an important role in stress response modulation [121]. This, subsequently, leads to AKT and mTORC1 activation, which results in activation of eukaryotic translation initiation factor 4E-binding protein 1 (4EBP1) and upregulation of the master eukaryotic translation switch eIF4E [121], which, in turn, initiates TLK1B translation. TLK1B phosphorylates NEK1, thereby triggering the ATR-CHK1-DDR signaling cascade [121]. Khalil and Benedetti suggested that activation of the DDR pathway through the TLK1-NEK1-ATR-CHK1 axis may be directly involved in the progression of CRPC [121].

Except for DDR activation, TLK1-NEK1 signaling affects other processes crucial for prostate cancer progression. Specifically, YAP/TAZ represent phosphorylation targets of NEK1, an important step in YAP/TAZ stabilization and nuclear accumulation [122,123]. In more detail, TLK1-NEK1-dependent phosphorylation of YAP/TAZ facilitates YAP/TAZ binding to TEAD or other transcription factors resulting in the transcription of pro-proliferative genes, a process fostering CRPC progression [121].

Although YAP may be necessary for prostate cancer progression, it may have a contradictory role upon radiation, as it was reported to enhance apoptosis mediated by early growth response protein 1 (EGR-1) [124]. EGR-1 is a well-known transcription factor, which together with *MYC*, *TP53* and *PTEN* constitute functionally important target genes of the Notch signaling pathway [125]. Mechanistically, it was demonstrated that upon IR, EGR-1/YAP1 complexes may transcriptionally activate the pro-apoptotic BAX gene [124]. Taken together, TLK-NEK signaling is another point where the DDR may directly converge with the Hippo and Notch pathways in prostate cancer.

### 5.5. Direct Hippo–Notch Pathway Crosstalk

A direct connection between the Hippo and Notch signaling pathways in the context of several human cancer types, including prostate, was recently identified, via the tumor suppressor RASSF1A [126]. RASSF1A is an established activator of the Hippo pathway, which is found epigenetically inactivated in a plethora of tumor types [127]. In prostate cancer, RASSF1A has been reported to be silenced in approximately 70% of patient tumors [128], which correlates with increased levels of the Notch downstream effector HES1 [126]. This reciprocal correlation between RASSF1A and HES1 was evident in the vast majority of human cancers, implying that the reported RASSF1A–HES1 interplay may be conserved among different tumor types [126]. Mechanistically, it was demonstrated that RASSF1A stabilizes the E3 ligase RNF4, which targets HES1 for proteasome-dependent degradation, thus preventing the expression of HES1-dependent proliferation and cancer stemness genes [126]. In contrast, in the absence of RASSF1A, RNF4 reduces its affinity with HES1, which remains stable regardless of upstream Notch signals or the use of Notch inhibitors [126], indicating that Notch signaling blockade at the receptor level (e.g., with γ-secretase inhibitors) may be fruitful only in the presence of RASSF1A in several cancer types, including prostate.

### 5.6. Potential Therapeutic Implications

Several components of the Hippo and Notch pathways have emerged as auspicious targets for the treatment of various types of cancer, including prostate [16,129] (Table 1). Crizotinib is a known inhibitor of anaplastic lymphoma kinase (ALK), which is a potential Hippo pathway regulator [130] and is found upregulated in prostatic small carcinoma [131]. Notably, it has been shown that crizotinib treatment activates ATM in response to oxidative DNA damage in other tumor types, such as gastric tumors [132]. Moreover, data from clinical trials provide evidence that focal adhesion kinase (FAK) inhibitors abrogate YAP activation in prostate cancer [133]. FAK is required for promoting DNA damage repair in *KRAS* non-small cell lung cancer (NSCLC) cells and FAK inhibitors augment the cytotoxic effects of IR by increasing DNA damage [134]. Additionally, mahanine, which belongs in the DNA methyltransferase inhibitor (DNMTi) family, induces proteasomal degradation of DNMT1 and DNMT3B via AKT inactivation in prostate cancer cells, thereby promoting RASSF1A promoter demethylation and restoration of its normal expression [135].

Apart from the above inhibitors which are clearly involved with the DDR pathway and have an already verified role against prostate tumorigenesis, there are additional Hippo/Notch pathway inhibitors with promising anti-tumorigenic effects, which however, have not been demonstrated in the prostate context yet. The FDA-approved drug verteporfin is widely used for YAP–TEAD complex inhibition, potentially through upregulation of the YAP chaperon 14-3-3σ, which maintains YAP in the cytoplasm and targets it for proteasomal degradation [129]. Treatment of breast cancer cells with verteporfin promoted apoptosis through BCL-2 decrease and caspase-9 and PARP cleavage [140]. Notably, verterporfin has also been found to inhibit DNA repair and promote apoptosis following IR in glioma cells [136]. Along the same lines, the tyrosine kinase inhibitor dasatinib, which promotes YAP/TAZ proteasomal degradation, induces DNA damage and impairs DNA repair signaling pathways, leading to p21-dependent senescence with loss of TAZ and CHK1 in NSCLC cells [137]. Moreover, statins are 3-hydroxy-3-methylglutaryl coenzyme A (HMG-CoA) reductase inhibitors which induce YAP/TAZ sequestration in the cytoplasm [129]. By inhibiting HMG-CoA, pitavastatin, a member of the statin family, delayed the DSB repair process and induced cellular senescence following IR in melanoma tumors [138]. Finally, GSIs that target the Notch pathway by preventing production of the Notch intracellular domain seem to be also implicated with the DDR pathway in melanoma cells [139]. In that setting, administration of the RO4929097 GSI inhibited IR-induced DDR [139].

As already mentioned, Hippo and Notch pathway components have been strongly associated with stemness in prostate cancer. Interestingly, it was reported that uncontrollable expression of YAP/TAZ may lead to CRPC by promoting stemness in prostate cancer cells [44]. There is evidence that prostate cancer stem cells (PCSCs) proliferate at a lower frequency compared to non-stem cells, thus manifesting increased survival to the cytotoxic effects of the DDR inducer etoposide [141]. In addition, PCSCs are known for their important contribution to radioresistance via several mechanisms, including increased DNA repair [142]. Specifically, radioresistant PCSCs exhibit increased phosphorylation of CHK2 leading to a prolonged cell-cycle arrest, as well as increased AKT activity resulting in enhanced DNA repair through NHEJ mediated by the PI3K signaling axis [142]. It is, thus, clear that prostate cancer stemness, regulated by the Hippo or Notch pathway, may confer considerable resistance to chemotherapy and radiotherapy via modulating aspects of the DDR pathway. The emerging role of PCSCs in prostate tumorigenesis and response to therapy renders them a promising therapeutic target.

## 6. Discussion

Prostate cancer, together with breast and ovarian cancer, belongs to the class of hormone-dependent cancers, which are the major cause of cancer incidence worldwide [1]. Despite the fact that ADT is an effective type of treatment in the early stage of prostate cancer, most patients with prostate cancer progress from an androgen-dependent to a castration-resistant type of disease for which there is no efficient therapy as yet. Therefore, developing novel therapeutic strategies for CRPC patients, by shedding light on the molecular pathways involved in this process, is of paramount importance. In this review, we recapitulate evidence regarding the interplay of the major developmental Hippo and Notch signaling pathways with the DDR in the prostate cancer context. This demonstrates the pleiotropic nature of several pathway components and additionally highlights which processes may be crucial for prostate cancer development. Targeting important molecular ‘hubs’ where the Hippo, Notch and DDR pathways converge, such as the AR, ERG, AKT and TLK/NEK, may yield productive therapeutic approaches in prostate cancer.

In prostate, among other types of cancer, cellular senescence may be promoted through activation of the DDR pathway, following treatment with radiation and/or certain chemotherapeutics [143,144]. Cellular senescence is a hallmark of aging triggered by various stress stimuli, defined by a chronic and generally irreversible cell-cycle arrest [145,146]. As such, it has been characterized as a natural barrier to cancer development and progression [147]. DNA damaging agents, such as poly (ADP-ribose) polymerase1 (PARP) inhibitors have been found to induce permanent p53-independent therapy-induced senescence in prostate cancer [148]. However, ADT may rather induce a reversible type of senescence which lacks the characteristics of DNA damage [148]. Along those lines, escape from senescence has been found to be an important mechanism contributing to tumor progression [145,149,150]. Interestingly, although several potential links with developmental pathways remain to be established, Notch signaling has been found associated with cellular senescence in prostate cancer, as upon PTEN loss, Notch pathway inhibition leads to senescence [78]. Moreover, in prostate epithelial cells, PTEN loss promotes activation of the DDR pathway in response to replication stress, progressively leading to cellular senescence [151]. Of note, potentially harmful senescent cells can be selectively targeted and eliminated by a class of compounds collectively referred to as senolytics [152,153]. It remains to be explored whether natural anti-cancer or anti-oxidative compounds [154,155] may additionally act as senolytic agents.

Deregulation of signaling pathways during tumorigenesis frequently results in escalating oncogenic stress serving as the driving force of DDR, which indicates that the integrity of signal transduction is cardinal in maintaining genome stability [156]. Deregulated Hippo and Notch pathways have been found to intersect with the DDR in a plethora of clinical settings; however, the relevance of those interactions in prostate tumorigenesis remains to be confirmed. For example, RASSF1A was recently shown to co-localize with translocated ribosomal DNA (rDNA) breaks at the nucleolar periphery in a 53BP1-mediated manner, thus promoting ATM signaling [157]. RASSF1A loss contributes to rDNA breaks and limits cell survival following rDNA damage, a finding which may also apply in the prostate context [157].

The association of ECM with the Hippo and Notch pathways appears to be important for cancer progression. In contrast to the normal “soft” environment in which cells are subjected to low mechanical forces frequently accompanied by reduced levels of YAP/TAZ [158], during cancer development many alterations occur in the tissue architecture. Those changes include the recruitment of stromal cells, such as cancer-associated fibroblasts (CAFs) or tumor-associated macrophages (TAMs) [159], which may modify the mechanical forces and promote ECM stiffening, which in turn activates YAP/TAZ and favors oncogenic proliferation and invasive behavior [60,160]. Of note, a mechanical interplay has been identified between YAP/TAZ and Notch signaling in the corneal epithelium [161]. Specifically, inactivation of Notch signaling has been found to promote an inflammatory response accompanied by DNA damage in corneal epithelial cells, ultimately leading to chronic inflammation, ECM remodeling and fibrosis [161]. This, in turn, activates YAP/TAZ mechanotransduction [162] promoting corneal squamous cell metaplasia (CSCM) [161]. All the above highlight the interconnection of the Hippo and Notch pathways with ECM, which has a potential role in prostate cancer progression that remains to be explored.

To delineate the mechanistic aspects of prostate cancer, the generation and optimization of advanced in vitro model systems recapitulating the complex and heterogeneous processes of prostate carcinogenesis, is of great importance. The first steps towards that direction have already been made through the generation of patient-derived organoids from several tissue types [163,164,165,166]. To that end, prostate organoids have already been generated directly from prostate cancer patients, potentially serving as powerful drug screening platforms with existing or novel therapeutic agents at the patient level [167,168,169,170,171].

As mentioned above, aspects of the Hippo/Notch and DDR interplay appear to hold an important role in prostate cancer development and progression. Those pathways separately control cancer cell proliferation, migration and invasion, and importantly, there are distinct molecular ‘hubs’ where their properties converge. Hence, rather than focusing on the pathways as separate entities, understanding the complex interactions between their key components is likely to contribute to our overall understanding of prostate tumorigenesis and allow for the design and implementation of efficient therapeutic strategies at a personalized level.

## Figures and Tables

**Table 1:** Therapeutic interventions potentially modulating the Hippo/Notch/DDR interplay.

Drug	Target	Effect	Implication with DDR Pathway
Crizotinib	Hippo Pathway	Inhibition of ALK	ATM phosphorylation and activation in response to oxidative DNA damage [132]
FAK inhibitors	Hippo Pathway	Abrogation of YAP activity	Induction of senescence and activation of DNA damage pathways [134]
DNMT inhibitors	Hippo Pathway	Demethylation of RASSF1A promoter	Mahanine: Inactivation of AKT, proteasomal degradation of DNMT1 and DNMT3B, demethylation and restoration of RASSF1A normal expression [135]
Verteporfin	Hippo Pathway	Induction of YAP sequestration in cytoplasm and inhibition of YAP–TEAD interaction	Inhibition of DNA repair and apoptosis following IR [136]
Dasatinib	Hippo Pathway	Induction of YAP/TAZ proteasomal degradation	Induction of DNA damage and senescence [137]
Statins	Hippo Pathway	Induction of YAP sequestration in cytoplasm	Pitavastatin: Inhibition of DNA repair and induction of senescence in vitro and in vivo [138]
GSIs	Notch Pathway	Inhibition of NICD production	RO4929097 + Radiation: Inhibition of the DDR pathway [139]

## Data Availability

Not applicable.
